# Analysis of Common Alpha-Globin Gene Abnormalities and Their Effects as Genetic Modifiers in Thai Children With β-Globin Gene Abnormalities

**DOI:** 10.1155/anem/9933808

**Published:** 2025-03-17

**Authors:** Sethapong Lertsakulbunlue, Boonchai Boonyawat, Chanchai Traivaree, Apichat Photia

**Affiliations:** ^1^Department of Pharmacology, Phramongkutklao College of Medicine, Bangkok 10400, Thailand; ^2^Division of Medical Genetics, Department of Pediatrics, Phramongkutklao Hospital and Phramongkutklao College of Medicine, Bangkok 10400, Thailand; ^3^Division of Hematology, Department of Pediatrics, Phramongkutklao Hospital and Phramongkutklao College of Medicine, Bangkok 10400, Thailand

**Keywords:** alpha-globin gene mutation, beta-thalassemia, genetic modifiers, pediatrics

## Abstract

Beta-thalassemia exhibits a broad phenotypic range influenced by the severity of *HBB* mutation and various genetic modifiers. One of the most essential modifiers is the coinheritance of α-globin gene mutation. Nevertheless, the understanding of these α-globin variations' impact on beta-thalassemia is lacking among pediatric patients. This study investigated the impact of common α-globin gene mutations on clinical phenotype and hematological parameters in 122 Thai children with either β-thalassemia diseases or carriers recruited from Phramongkutklao Hospital, a major thalassemia center. Clinical characteristics, transfusion history, and hematological parameters were recorded, with molecular testing for common α-globin deletions and Hb CS mutations. The cohort included 8 homozygous β-thalassemia, 55 β-thalassemia/Hb E, 18 homozygous Hb E, 26 heterozygous Hb E, and 15 heterozygous β-thalassemia children. Coinheritance of α-globin mutations was less frequent in β-thalassemia diseases (6 of 63) than in β-thalassemia traits (25 of 59) (*p* < 0.001), indicating a potential modifier effect that reduces severity. Among β-thalassemia/Hb E patients, single α-globin deletions or Hb CS mutations were linked with lower Hb E, MCV, and MCH. Similarly, in both β-thalassemia and Hb E traits with α-globin gene mutation had significantly lower MCV, MCH and Hb E levels (only in the Hb E trait) and elevated RDW. Moreover, lower hematocrit and hemoglobin in these carriers were noted in cases coinherited with deletional Hb H disease initially undiagnosed by Hb typing. In conclusion, the diagnostic value of hematological parameters and Hb typing in identifying common α-globin mutations in pediatric β-thalassemia patients were highlighted. Hematological parameters are vital indicators that may prompt genetic screening to confirm α-globin abnormalities, supporting improved diagnosis and management of complex αβ-thalassemia syndromes.

## 1. Introduction

Thalassemia is a group of inherited monogenic hemolytic anemias characterized by reduced or absent synthesis of globin chains, composed of alpha- and beta-thalassemia [1]. Mutations in the *HBB* gene cause beta-thalassemia (β-thalassemia). Patients may exhibit varied clinical presentations such as age of onset, transfusion requirements, and severity of hepatosplenomegaly. Individuals with one mutated *HBB* allele are classified as “Thalassemia Trait” (TT), which are usually asymptomatic. Those with two null-allele *HBB* mutations, including β^0^/β^0^ and β^0^/B^E^ are classified as “Thalassemia Major” (TM), which typically requires regular blood transfusions and the term β-thalassemia Intermedia (TI), which the disease severity lies between TT and TM [2]. However, phenotypic heterogeneity in β-thalassemia is not fully explained by variation of *HBB* gene mutations alone, leading to diagnostic and therapeutic challenges [3, 4].

Several genetic factors modify the phenotype of β-thalassemia. One of the essential primary modifiers of β-thalassemia severity is the coinheritance of the alpha-globin gene, including *HBA1* and *HBA2* variants [5–7]. Alpha-thalassemia is predominantly a health burden in Southeast Asia [8]. In Thailand, the prevalence of α-thal-1 and α-thal-2 is estimated at 2.5%–10% and 10%–20%, respectively [9, 10]. Moreover, the prevalence of α-thalassemia variants in Thailand showed significant regional variation, with Southeast Asian (SEA) α-thal-1 deletion ranging from 3.1% to 21.0% and α-thal-2 deletion including both 3.7 kb and 4.2 kb deletions reaching up to 25.1% in the northeastern region. While Hb CS prevalence ranged from 4.5% to 5.6%, the THAI α-thal-1 deletion type was rare at 0.11% [11, 12].

Despite Thailand being an endemic region for thalassemia, the impact of α-globin gene mutations on the phenotype modification of β-thalassemia remains underexplored, especially in children. This study aimed to investigate the effects of α-globin gene mutations on clinical phenotype and hematological parameters in pediatric patients with homozygous β-thalassemia, β-thalassemia/Hb E disease, β-thalassemia carriers, homozygous Hb E, and Hb E traits at Phramongkutklao Hospital, a tertiary care center for thalassemia patients across Thailand. These findings could provide valuable insights to improve the diagnosis and management of complex αβ-thalassemia syndromes, particularly given that routine molecular testing for α-globin gene mutations is not yet widely available.

## 2. Materials and Methods

### 2.1. Study Design and Subjects

This study initially included 132 individuals diagnosed with *HBB* mutations, classified as either carriers or diseases, from January 1, 2023, to January 1, 2024. Participants were recruited from the Hematology Clinic at the Department of Pediatrics, Phramongkutklao Hospital, Bangkok, Thailand. A total of 10 patients with transfusion dependency were excluded since their hematological parameters were not recorded either during baseline blood tests or in a steady state. Similarly, patients with thalassemia traits were excluded if their iron status was unavailable or if they had iron deficiency without hematological parameters obtained after treatment for the deficiency. Thus, the present study included a total of 122 participants.

Written informed consent and assent forms were obtained from all participants; for minors, their parents or guardians provided consent before enrollment. The study protocol received approval from the Institutional Review Board of Phramongkutklao Hospital and Phramongkutklao College of Medicine (approval no. S044b/67_Exp). It adhered to the ethical principles outlined in the Declaration of Helsinki (1975) and its subsequent revisions. All patients were diagnosed before the age of 18. Patients with homozygous β-thalassemia and β-thalassemia/Hb E were clinically classified as severe transfusion-dependent TM or TI based on criteria including age at presentation, average steady-state hemoglobin level, and transfusion frequency history, as previously described [13]. Demographic information, physical examination findings, blood transfusion history and hematological parameters—including complete blood count, red blood cell index, and Hb typing—and laboratory test results were recorded for all patients.

### 2.2. Hematological Parameters

Hematological analyses were conducted using the Coulter HMX Automated Hematology Analyzer (Beckman Coulter Corporation, Brea, CA, USA). Hemoglobin profiles and fetal hemoglobin (HbF) concentrations were determined using Capillary Electrophoresis (Minicap system, Sebia, Parc Technologique Leonard de Vinci, Evry Cedex, France), as previously described [14].

### 2.3. Molecular Methods

Peripheral blood EDTA samples were collected from all participants after obtaining informed consent. Genomic DNA was extracted from peripheral blood lymphocytes following a standard protocol. Initially, *HBB* gene mutations were identified using the multiplex amplification refractory mutation system (M-ARMS) to detect seven common mutations in Thailand, as previously described [15]. In cases where the *HBB* gene mutations were not identified through M-ARMS, further investigation was conducted using direct DNA sequencing to detect less common mutations, covering all three exons and the exon-intron junctions. In addition, for β-thalassemia alleles that remained uncharacterized by M-ARMS and sequencing methods, gap-PCR was employed to screen for the 3.4 kb deletion known to be present in Thai populations [15].

Molecular analysis of *HBA1* and *HBA2* gene mutations was conducted to detect common α-globin mutations in the Thai population [16]. Initially, multiplex gap PCR was employed to characterize common α-globin gene deletions prevalent in Southeast Asia, including α-thal-1 (SEA [−^SEA^] and THAI [−^THAI^] deletions) and α-thal-2 (3.7 kb [−α^3.7^] and 4.2 kb [−α^4.2^] deletions). Subsequently, M-ARMS was utilized to detect Hb CS, Thailand's most common nondeletional α-globin gene mutation [16].

### 2.4. Statistical Analysis

Baseline characteristics and hematological parameters were reported as the frequency and percentage for categorical data and as mean with the standard deviation or median with interquartile range for continuous data, as appropriate. The distribution of quantitative variables was assessed using the Shapiro–Wilk test. Continuous data were compared between two groups using the unpaired *t*-test for parametric distributions and the Mann–Whitney *U*-test for nonparametric distributions. Categorical variables were analyzed using the chi-square test or the Fisher exact test, as appropriate. For comparisons involving more than two groups, one-way ANOVA was used for parametric distributions, and the Kruskal–Wallis test was used for nonparametric distributions. Statistical analyses were conducted using SPSS Version 29 (IBM Corporation, Armonk, NY, USA), with *p* values < 0.05 considered significant.

## 3. Results

### 3.1. Characteristics of Participants

A total of 122 patients were enrolled in this study.  Table 1 summarizes the demographic characteristics and the findings of common *HBA1* and *HBA2* mutations. The majority of participants were male (*n* = 74, 65.5%). The mean ages at diagnosis were 0.5 ± 0.3, 2.1 ± 1.9, 4.2 ± 3.4, 11.4 ± 6.7, and 6.2 ± 5.5 years for individuals with homozygous β-thalassemia, β-thalassemia/Hb E, homozygous Hb E, heterozygous Hb E, and heterozygous β-thalassemia, respectively.

The cohort includes 8 patients with homozygous β-thalassemia, 55 with β-thalassemia/Hb E, 18 with homozygous Hb E, 26 with heterozygous Hb E, and 15 with heterozygous β-thalassemia. Among the 71 alleles identified in patients with β-thalassemia and β-thalassemia/Hb E diseases, *HBB* mutations included 7 severe β^+^-thalassemia mutations (6 cases of IVS-I-5 (G> C) and 1 case of codon 19 (A > G)). The 64 remaining alleles, classified as β^0^-thalassemia, comprised 31 cases of codon 41/42 (−TCTT), 22 cases of codon 17 (A > T), 5 cases of IVS-II-654 (C > T), 4 cases of IVS-I-1 (G > T), and 2 cases of codon 71/72 (+A) mutation.

**Figure 1:**
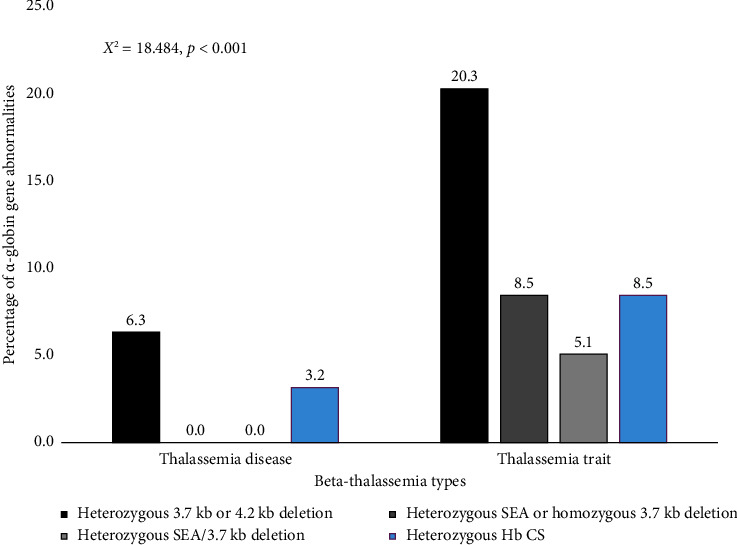
Alpha-globin gene abnormalities stratified by beta-thalassemia phenotypes.

Among common α-thalassemia mutations, the 3.7 kb deletion (−α^3.7^) was the most frequent, identified in 16 patients (13.1%). The Southeast Asian deletion (−^SEA^) was observed in 4 patients (3.3%), while the 4.2 kb deletion (−α^4.2^) was detected in only 1 patient. Notably, 3 patients (2.5%) exhibited both the 3.7 kb and SEA deletions. The heterozygous Hb CS mutation was found in 7 patients (5.7%). Furthermore, the frequency of *HBA1* and *HBA2* mutations was significantly lower in patients with β-thalassemia disease compared with β-thalassemia carriers (*p* < 0.001), as illustrated in Figure 1.

### 3.2. Alpha-Globin Gene Abnormalities and Genotypic–Phenotypic Correlation in β-Thalassemia

Table 2 highlights the clinical and laboratory profiles of β-thalassemia/Hb E patients, stratified by α-globin gene abnormalities, revealing significant variations in hematocrit, MCV, MCH, and Hb E levels across different genetic profiles. Patients with normal α-globin genes exhibited median hematocrit and MCV levels of 24.8% and 62.8 fl, respectively, while those with a single −α^3.7^ deletion had similar hematocrit levels but lower MCV (56.5 fl). In contrast, patients with the Hb CS mutation showed markedly lower MCV (53.3 fl) levels (*p* < 0.001). The MCH was significantly lower in patients with Hb CS (16.1 pg) compared with those without deletions (20.4 pg), with a *p*-value of < 0.001. In addition, Hb E levels were reduced in patients with the −α^3.7^ deletion (32.6%) and Hb CS mutation (31.7%) compared with those with normal α-globin genes (45.7%).

Clinical and laboratory profiles of homozygous Hb E stratified by α-globin gene abnormalities are presented in Table 3. The hematocrit, MCV, MCH, and MCHC values remained consistent across the genetic groups, indicating that specific α-globin mutations did not significantly influence these parameters. However, Hb E levels varied notably among the groups. Patients with two −α^3.7^ deletions had the lowest Hb E levels, averaging 74.2%, while those with the heterozygous Hb CS mutation showed slightly higher but still reduced levels at 74.7%. In contrast, patients with normal α-globin genes had a median Hb E level of 82.1%, and the highest levels were observed in individuals with a single −α^3.7^ deletion (median: 84.7%). These findings suggest that while most hematological parameters remain unaffected, specific α-globin gene abnormalities, particularly double deletions and the Hb CS mutation, are associated with reduced Hb E levels.

**Figure 2:**
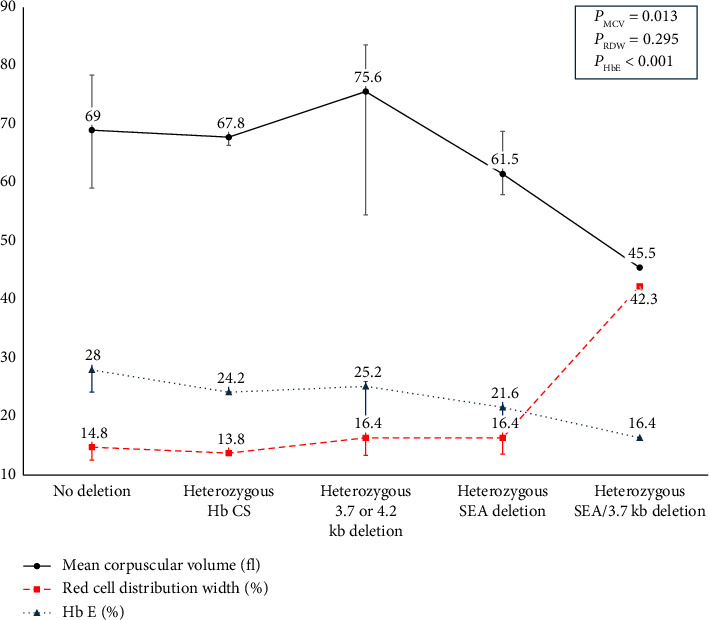
The hematological parameter with observable trend among heterozygous hemoglobin E stratified by α-globin gene abnormalities.

Table 4 presents the clinical and laboratory profiles of 26 heterozygous Hb E patients categorized by α-globin gene abnormalities. The cohort includes four individuals with single deletions (three with −α^3.7^ deletions and one with −α^4.2^ deletion), four individuals with double deletions (all with the −^SEA^), one with −α^3.7^ deletion combined with the −^SEA^ deletion, and three with heterozygous Hb CS mutations. A progressive decline in MCV, MCH, and Hb E levels was observed with increased deletions and a corresponding rise in RDW, indicating greater red blood cell size variability (Figure 2).

**Figure 3:**
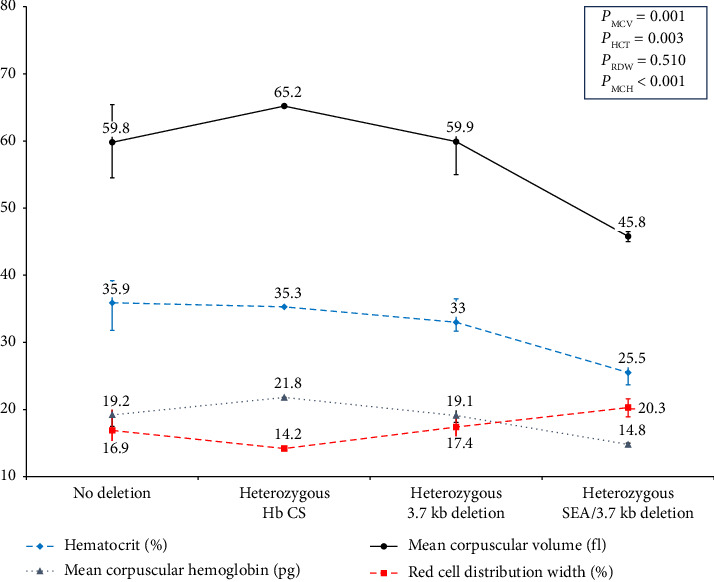
The hematological parameter with observable trend among heterozygous β-thalassemia stratified by α-globin gene abnormalities.

Clinical and hematological profiles of homozygous β-thalassemia patients were demonstrated in Table 5; neither common α-globin deletions nor Hb CS was identified in these patients.  Table 6 summarizes the clinical and hematological parameters of 15 heterozygous β-thalassemia patients stratified by α-globin gene abnormalities. Among them, 4 had the −α3.7 deletion, 2 had three α-globin gene deletions (one −α^3.7^ and one −^SEA^ deletion, consistent with deletional Hb H disease), and 1 had a heterozygous Hb CS mutation. In β-thalassemia trait patients, increasing α-globin gene deletions were associated with a gradual decline in MCV, MCH, hematocrit, and hemoglobin levels, along with an increase in RDW, as shown in Figure 3.

## 4. Discussion

This study examined the clinical and hematological profiles in individuals with various forms of β-thalassemia and Hb E and, remarkably, how different α-globin gene abnormalities affect these profiles among pediatric patients. Furthermore, unlike previous relevant studies, as Thailand is endemic for Hb E, this study provides valuable insights into this group. The findings underscore the significant impact of α-globin gene deletions and Hb CS mutations on key hematological parameters, such as hematocrit, hemoglobin levels, MCV, MCH, and RDW.

Coinheritance of α-thalassemia had been observed in 25/59 (42.4%) of the participants who were carriers of either β-thalassemia or Hb E, including homozygous Hb E, heterozygous Hb E, and heterozygous β-thalassemia. Contrarily, only 6/63 (9.5%) coinheritance of α-thalassemia had been observed among those within the transfusion-dependent β-thalassemia group, including homozygous β-thalassemia and β-thalassemia/Hb E. Coinheritance of α-thalassemia has been identified as a significant genetic factor contributing to ameliorating TM to TI in Sri Lanka, India, Italy, and Pakistan, with reported occurrences in 9/50 (18.0%), 16/73 (21.9%), 10/74 (19.5%), and 13/63 (20.6%) of TI patients, respectively [5, 17–19]. In addition, studies in Cyprus and Sardinia have shown that the coinheritance of one or two α-globin gene deletions in homozygous β-thalassemia patients is one of the most significant factors in mitigating the severity of the disease [20, 21].

The current study included only pediatric patients with β-thalassemia. All patients with β-thalassemia diseases carried only severe β^+^ or β^0^ mutations. Consequently, symptoms in these patients manifested early and were generally severe. This finding confirms that the primary genetic factor influencing the phenotype of patients is the severity of HBB mutations, consistent with observations from previous studies [14]. Nonetheless, the coinheritance of common α-globin gene mutations was less prevalent among individuals with β-thalassemia disease than those with β-thalassemia trait. Notably, no homozygous β-thalassemia patients in this study carried α-globin gene variants. In contrast, the prevalence of these variants in the β-thalassemia trait is comparable with that of the general Thai population [9, 12]. This finding suggests that the coinheritance of α-globin gene abnormalities may attenuate the severity of β-thalassemia disease.

Similarly, the present study observed a cohort of 55 β-thalassemia/Hb E patients. Notably, none had two or more α-globin gene deletions, suggesting a potential effect modification that may result in a milder disease. This finding aligns with previous studies suggesting that two or more α-globin gene deletions may reduce the clinical severity of compound heterozygous and homozygous β-thalassemia [5, 6, 22]. Moreover, in β-thalassemia/Hb E patients with either a single α-globin gene deletion or an Hb CS mutation were associated with lower MCV and MCH levels. Although not statistically significant due to limited statistical power, the median Hb E levels among β-thalassemia/Hb E patients with single α-globin gene deletion or Hb CS mutation were approximately 32.6 and 31.7, respectively, compared with a median Hb E of 45.7 in patients without such mutations. This finding aligns with previous studies showing that Hb E constitutes between 30% and 70% of total hemoglobin in β-thalassemia/Hb E patients, with α-globin gene deletions contributing to lower Hb E levels [23]. However, due to the disease's wide range of Hb E levels depending on the β-thalassemia mutation types and age group, no specific cutoff values are recommended for additional α-globin gene testing [23].

The present study included 18 homozygous Hb E patients and observed that either α-globin gene deletions or Hb CS mutation may not significantly affect hematological parameters. However, two α-globin gene deletion or Hb CS mutation reduces the median Hb E levels from over 80%–74%. This is consistent with prior research on α-globin deletions in homozygous Hb E patients, showing significantly lower Hb E levels in those with the α^0^-thalassemia (SEA-type deletion) trait (87.8 ± 5.2) compared to those without the α^0^-thalassemia trait (92.35 ± 2.2) [24]. In comparison, a study in Thailand revealed relatively lower Hb E levels only among those with three α-globin deletions [23]. Furthermore, patients with the α^0^-thalassemia (SEA-type deletion) trait also demonstrated higher HbA_2_ levels [24]. Thus, a reduction in Hb E levels coupled with elevated HbA_2_ levels in homozygous Hb E patients may indicate the need for further molecular testing for common α-globin gene mutations.

In the heterozygous Hb E and heterozygous β-thalassemia groups, two or three α-globin gene deletions result in significant changes in hematocrit, hemoglobin, MCV, MCH, RDW, and Hb E levels. Particularly among the heterozygous Hb E group, a Hb E percentage below approximately 24%–26% might be an essential clue of the coinheritance of α-thalassemia [23, 25, 26]. Additionally, in this study, heterozygous Hb E cases with three α-globin gene deletions—indicative of deletional Hb H disease—demonstrated reduced MCV and MCH, Hb E level below 20%, and a marked increase in RDW to over 40%, whereas RDW generally remains within normal ranges in minor thalassemia [23, 27]. Similarly, in β-thalassemia traits, reductions in hematocrit and hemoglobin were observed only in cases with coinheritance of deletional Hb H disease. Coinheritance of β-thalassemia trait and deletional Hb H disease can complicate diagnosis, as this combination may suppress Hb H expression, leading to potential misdiagnosis, specifically Hb typing with high-performance liquid chromatography alone [28, 29]. Therefore, molecular analysis of α-globin genes is recommended in cases of hemolytic anemia where Hb typing indicates heterozygous Hb E or β-thalassemia trait, particularly when MCV, MCH, and Hb E levels are lower, and RDW is elevated than expected.

This study provides valuable insights into complex αβ-thalassemia syndromes in the pediatric population. A key strength of this research is the inclusion of Hb E groups, which distinguishes it from previous studies. The findings underscore the importance of comprehensive genetic screening in both β-thalassemia carriers and individuals with β-thalassemia diseases, as the type and number of α-globin gene abnormalities can significantly impact disease severity and management strategies. This research contributes to a deeper understanding of the genotype–phenotype correlation in β-thalassemia. In addition, incorporating hematological parameters such as hematocrit, hemoglobin, MCV, MCH, RDW, and Hb E levels may offer important insights for further screening of complex αβ-thalassemia syndromes, particularly in pregnant women, to help prevent additional complications [30].

This study has several limitations that should be acknowledged. The participants were drawn from Phramongkutklao Hospital, a tertiary care center, which may limit the generalizability of the findings, as the prevalence of complex αβ-thalassemia syndromes might differ in other settings. In addition, the study included subgroup analyses of different β-thalassemia groups, resulting in relatively low statistical power for less common mutation groups. Despite this, trends in hematological parameters were still observable. Therefore, further research is needed to explore the long-term clinical implications of these findings, particularly in larger and more diverse patient populations. Moreover, potential interactions between α-globin gene abnormalities and other genetic or environmental factors should be investigated to provide a more comprehensive understanding of thalassemia pathophysiology. In addition, other potential factors, such as iron level, should be considered, as they may also influence these parameters [31, 32].

## 5. Conclusion

This study highlights the diagnostic value of hematological parameters and Hb typing in detecting coinheritance of α-globin deletions in pediatric patients with β-thalassemia diseases and carriers. Specifically, unexpectedly low Hb E levels, elevated RDW, and reduced hematocrit, hemoglobin, MCV, and MCH may serve as valuable indicators for further genetic screening to confirm α-globin gene abnormalities. These parameters provide essential guidance in identifying complex αβ-thalassemia syndromes, facilitating more accurate diagnosis and management. In addition, the observed roles of common α-globin gene deletions and Hb CS mutations in modulating β-thalassemia disease severity underscore the importance of understanding genotype–phenotype correlations to optimize care, improve outcomes, and enhance screening for complex αβ-thalassemia syndromes.

## Figures and Tables

**Table 1:** Demographic data and alpha-thalassemia mutations in beta-thalassemia patients (*n* = 122).

Characteristics	Homozygous β-thalassemia	β-thalassemia/Hb E	Homozygous Hb E	Heterozygous Hb E	Heterozygous β-thalassemia
	*n* (%)	*n* (%)	*n* (%)	*n* (%)	*n* (%)
Total	8 (100.0)	55 (100.0)	18 (100.0)	26 (100.0)	15 (100.0)
Gender					
Male	5 (62.5)	37 (67.3)	9 (50.0)	12 (46.2)	10 (66.7)
Female	3 (37.5)	18 (32.7)	9 (50.0)	14 (53.9)	5 (33.3)
Age (year) at diagnosis (mean ± SD)	0.5 ± 0.3	2.1 ± 1.9	4.2 ± 3.4	11.4 ± 6.7	6.2 ± 5.5
α-globin gene abnormalities					
Heterozygous 3.7 kb deletion	0	4 (7.3%)	4 (22.2%)	3 (11.5%)	4 (26.7%)
Heterozygous 4.2 kb deletion	0	0	0	1 (3.8%)	0
Heterozygoous SEA deletion	0	0	0	4 (15.4%)	0
Homozygous 3.7 kb deletion	0	0	1 (5.6%)	0	0
Heterozygous SEA/3.7 kb deletion	0	0	0	1 (3.8%)	2 (13.3%)
Heterozygous Hb CS	0	2 (3.6%)	1 (5.6%)	3 (11.5%)	1 (6.7%)
Normal	8 (100.0%)	49 (89.2%)	12 (66.7%)	14 (53.9%)	8 (53.3%)

Abbreviations: Hb CS, hemoglobin constant spring; Hb E; hemoglobin E; SD, standard deviation; SEA, Southeast Asian.

**Table 2:** Clinical and laboratory profile of β-thalassemia/Hb E stratified by α-globin gene abnormalities (*n* = 55).

β-thalassemia/Hb E	α-globin gene abnormalities			*p* value
	Deletional mutation		Nondeletional mutation	
	No deletion (*n* = 49)	Heterozygous 3.7 kb deletion (*n* = 4)	Heterozygous Hb CS (*n* = 2)	
Hemoglobin (g/dL)				
Median (min–max)	8.0 (6.0–9.6)	8.3 (7.4–8.9)	5.6 (3.5–7.7)	0.214
Hematocrit (%)				
Median (min–max)	24.8 (17.5–28.5)	25.7 (24.5–27.1)	17.7 (11.2–24.2)	0.175
Mean corpuscular volume (fl)				
Median (min–max)	62.8 (50.6–78.7)	56.5 (53.0–65.8)	53.3 (50.6–56.0)	< 0.001
Mean corpuscular hemoglobin (pg)				
Median (min–max)	20.4 (16.7–24.7)	18.6 (16.0–20.9)	16.1 (15.6–16.6)	0.003
Mean corpuscular hemoglobin concentration (g/dL)				
Median (min–max)	32.5 (29.5–35.5)	32.6 (31.8–33.2)	30.3 (29.7–30.8)	0.115
Red cell distribution width (%)				
Median (min–max)	28.6 (20.4–42.8)	32.6 (25.5–39.0)	31.7 (22.1–41.2)	0.991
Hb E (%)				
Median (min–max)	45.7 (29.3–66.1)	32.6 (25.5–39.0)	31.7 (22.1–41.2)	0.388
Treatment (*n* [%])				0.793
No treatment	2 (4.1)	0	0	
Blood transfusion	35 (71.4)	4 (100.0)	2 (100.0)	
Blood transfusion with splenectomy	12 (24.5)	0	0	
Blood transfusion frequency (*n* [%])				0.226
< 4 weeks	4 (8.2)	0	0	
4–6 weeks	36 (73.5)	2 (50.0)	1 (50.0)	
> 6 weeks	7 (14.3)	1 (25.0)	0	
When symptom presented	1 (2.0)	0	1 (50.0)	
Physical examination (*n* [%])				
Anemia	46 (93.9)	4 (100.0)	2 (100.0)	1.000
Jaundice	13 (26.5)	0	0	0.746
Hepatomegaly	41 (83.7)	4 (100.0)	1 (50.0)	0.393
Splenomegaly	37 (75.5)	4 (100.0)	1 (50.0)	0.404

Abbreviations: Hb CS, hemoglobin constant spring; Hb E, hemoglobin E.

**Table 3:** Clinical and laboratory profile of homozygous Hb E stratified by α-globin gene abnormalities (*n* = 18).

Homozygous Hb E	α-globin gene abnormalities				*p* value
	Deletional mutation			Nondeletional mutation	
	No deletion (*n* = 12)	Heterozygous 3.7 kb deletion (*n* = 4)	Homozygous 3.7 kb deletion (*n* = 1)	Heterozygous Hb CS (*n* = 1)	
Hemoglobin (g/dL)					
Median (min–max)	10.7 (9.8–12.1)	10.8 (10.5–11.0)	10.0	11.4	0.289
Hematocrit (%)					
Median (min–max)	32.0 (29.8–38.3)	32.1 (30.8–38.6)	28.2	35.9	0.394
Mean corpuscular volume (fl)					
Median (min–max)	58.8 (52.0–70.7)	54.3 (51.4–62.0)	50.3	58.8	0.513
Mean corpuscular hemoglobin (pg)					
Median (min–max)	18.9 (17.3–24.4)	18.4 (17.7–19.0)	17.8	18.7	0.506
Mean corpuscular hemoglobin concentration (g/dL)					
Median (min–max)	33.1 (31.5–36.1)	33.9 (28.6–35.3)	35.5	31.9	0.390
Red cell distribution width (%)					
Median (min–max)	19.4 (16.1–22.2)	17.8 (17.1–21.0)	17.9	19.9	0.792
Hb E (%)					
Median (min–max)	82.1 (70.6–87.9)	84.7 (81.9–90.5)	74.2	74.7	0.170

Abbreviations: Hb CS, hemoglobin constant spring; Hb E, hemoglobin E.

**Table 4:** Clinical and laboratory profile of heterozygous Hb E stratified by α-globin gene abnormalities (*n* = 26).

Heterozygous Hb E	α-globin gene abnormalities					*p* value
	Deletional mutation				Nondeletional mutation	
	No deletion (*n* = 14)	Heterozygous 3.7 kb or 4.2 kb deletion (*n* = 4)	Heterozygous SEA deletion (*n* = 4)	Heterozygous SEA/3.7 kb deletion (*n* = 1)	Heterozygous Hb CS (*n* = 3)	
Hemoglobin (g/dL)						
Median (min–max)	11.7 (9.9–14.0)	11.4 (9.9–11.7)	10.4 (9.3–11.1)	9.6	11.4 (9.9–12.0)	0.025
Hematocrit (%)						
Median (min–max)	36.0 (29.4–42.7)	34.0 (29.9–35.9)	32.6 (29.3–35.0)	28.4	33.3 (29.4–35.9)	0.014
Mean corpuscular volume (fl)						
Median (min–max)	69.0 (59.1–78.4)	75.6 (54.5–83.6)	61.5 (58.0–68.8)	45.5	67.8 (66.4–71.4)	0.013
Mean corpuscular hemoglobin (pg)						
Median (min–max)	23.3 (19.9–25.8)	24.9 (18.8–26.6)	20.1 (17.7–21.8)	15.4	23.2 (22.3–25.3)	0.004
Mean corpuscular hemoglobin concentration (g/dL)						
Median (min–max)	33.2 (31.7–35.6)	32.9 (31.8–35.5)	31.8 (30.3–33.6)	33.8	34.2 (33.7–35.4)	0.181
Red cell distribution width (%)						
Median (min–max)	14.8 (13.7–18.4)	16.4 (13.4–19.6)	16.4 (13.6–18.2)	42.3	13.8 (13.7–15.0)	0.295
Hb E (%)						
Median (min–max)	28.0 (24.2–30.6)	25.2 (19.0–26.1)	21.6 (18.5–23.9)	16.4	24.2 (24.2–26.3)	< 0.001

Abbreviations: Hb CS, hemoglobin constant spring; Hb E, hemoglobin E; SEA, Southeast Asian.

**Table 5:** Clinical and laboratory profile of homozygous β-thalassemia stratified by α-globin gene abnormalities (*n* = 8).

Homozygous β-thalassemia	α-globin gene abnormalities
	No deletion (*n* = 8)
Hemoglobin (g/dL)	
Median (min–max)	7.9 (4.7–8.6)
Hematocrit (%)	
Median (min–max)	20.6 (16.1–25.5)
Mean corpuscular volume (fl)	
Median (min–max)	73.2 (58.9–76.5)
Mean corpuscular hemoglobin (pg)	
Median (min–max)	25.2 (21.9–26.7)
Mean corpuscular hemoglobin concentration (g/dL)	
Median (min–max)	33.0 (32.1–37.2)
Red cell distribution width (%)	
Median (min–max)	19.7 (14.2–31.6)
Treatment (*n* [%])	
No treatment	0
Blood transfusion	2 (25.0)
Blood transfusion with splenectomy	6 (75.0)
Blood transfusion frequency (*n* [%])	
< 4 weeks	1 (12.5)
4–6 weeks	5 (62.5)
> 6 weeks	2 (25.0)
When symptom presented	0
Physical examination (*n* [%])	
Anemia	8 (100.0)
Jaundice	2 (25.0)
Hepatomegaly	8 (100.0)
Splenomegaly	5 (62.5)

**Table 6:** Clinical and laboratory profile of heterozygous β-thalassemia stratified by α-globin gene abnormalities (*n* = 15).

Heterozygous β-thalassemia	α-globin gene abnormalities				*p* value
	Deletional mutation			Nondeletional mutation	
	No deletion (*n* = 8)	Heterozygous 3.7 kb deletion (*n* = 4)	Heterozygous SEA/3.7 kb deletion (*n* = 2)	Heterozygous Hb CS (*n* = 1)	
Hemoglobin (g/dL)					
Median (min–max)	11.7 (10.2–12.2)	10.9 (10.0–12.3)	8.4 (7.4–9.3)	11.8	0.010
Hematocrit (%)					
Median (min–max)	35.9 (31.8–39.2)	33.0 (31.7–36.5)	25.5 (23.7–27.3)	35.3	0.003
Mean corpuscular volume (fl)					
Median (min–max)	59.8 (54.5–65.2)	59.9 (55.0–62.0)	45.8 (45.0–46.5)	65.2	0.001
Mean corpuscular hemoglobin (pg)					
Median (min–max)	19.2 (17.5–21.8)	19.1 (18.1–20.9)	14.8 (14.5–15.0)	21.8	< 0.001
Mean corpuscular hemoglobin concentration (g/dL)					
Median (min–max)	32.2 (29.6–43.1)	33.0 (31.5–34.0)	32.4 (31.2–33.5)	33.4	0.934
Red cell distribution width (%)					
Median (min–max)	16.9 (14.2–20.7)	17.4 (15.2–18.9)	20.3 (18.9–21.6)	14.2	0.510

Abbreviation: Hb CS, hemoglobin constant spring.

## Data Availability

The data used to support the findings of this study are available from the corresponding author upon reasonable request.

## References

[1] Taher A. T., Weatherall D. J., Cappellini M. D. (2018). Thalassaemia. *The Lancet*.

[2] Thein S. L. (2005). Genetic Modifiers of Beta-Thalassemia. *Haematologica*.

[3] Yamsri S., Singha K., Prajantasen T. (2015). A Large Cohort of β+-Thalassemia in Thailand: Molecular, Hematological and Diagnostic Considerations. *Blood Cells Molecules and Diseases*.

[4] Nuntakarn L., Fucharoen S., Fucharoen G., Sanchaisuriya K., Jetsrisuparb A., Wiangnon S. (2009). Molecular, Hematological and Clinical Aspects of Thalassemia Major and Thalassemia Intermedia Associated With Hb E-β-Thalassemia in Northeast Thailand. *Blood Cells Molecules and Diseases*.

[5] Perera S., Allen A., Silva I. (2019). Genotype-Phenotype Association Analysis Identifies the Role of α Globin Genes in Modulating Disease Severity of β Thalassaemia Intermedia in Sri Lanka. *Scientific Reports*.

[6] Saha D., Chowdhury P. Kr, Panja A. (2022). Effect of Deletions in the α-Globin Gene on the Phenotype Severity of β-Thalassemia. *Hemoglobin*.

[7] Winichagoon P., Fucharoen S., Chen P., Wasi P. (2000). Genetic Factors Affecting Clinical Severity in β-Thalassemia Syndromes. *American Journal of Pediatric Hematology*.

[8] Hockham C., Ekwattanakit S., Bhatt S. (2019). Estimating the Burden of α-Thalassaemia in Thailand Using a Comprehensive Prevalence Database for Southeast Asia. *Elife*.

[9] Fucharoen S., Winichagoon P. (1987). Hemoglobinopathies in Southeast Asia. *Hemoglobin*.

[10] Wasi P., Pootrakul S., Pootrakul P., Pravatmuang P., Winichagoon P., Fucharoen S. (1980). Thalassemia in Thailand. *Annals of the New York Academy of Sciences*.

[11] Chaibunruang A., Sornkayasit K., Chewasateanchai M., Sanugul P., Fucharoen G., Fucharoen S. (2018). Prevalence of Thalassemia Among Newborns: A Re-Visited After 20 Years of a Prevention and Control Program in Northeast Thailand. *Mediterranean Journal of Hematology and Infectious Diseases*.

[12] Leckngam P. (2023). Thalassemia and Hemoglobinopathies in Thailand: A Systematic Review. *Journal of Health Science and Alternative Medicine*.

[13] Ho H., Luo W. (1998). Thein. Beta‐Thalassaemia Intermedia: Is it Possible Consistently to Predict Phenotype From Genotype?. *British Journal of Haematology*.

[14] Traivaree C (2018). Genotype–Phenotype Correlation Among Beta-Thalassemia and Beta-Thalassemia/HbE Disease in Thai Children: Predictable Clinical Spectrum Using Genotypic Analysis. *Journal of Blood Medicine*.

[15] Traivaree C., Boonyawat B., Monsareenusorn C. (2014). Molecular Analysis of Beta-Globin Gene Mutations Among Thai Beta-Thalassemia Children: Results From a Single Center Study. *The Application of Clinical Genetics*.

[16] Boonyawat B., Photia A., Monsereenusorn C., Rujkijyanont P., Traivaree C. (2017). Molecular Characterization of Hb H and AEBart’s Diseases in Thai Children: Phramongkutklao Hospital Experiences. *Medical Journal of the Medical Association of Thailand*.

[17] Panigrahi I., Agarwal S., Pradhan M., Choudhry D. R., Choudhry V. P., Saxena R. (2006). Molecular Characterization of Thalassemia Intermedia in Indians. *Haematologica*.

[18] Camaschella C., Mazza U., Roetto A. (1995). Genetic Interactions in Thalassemia Intermedia: Analysis of β‐Mutations, α‐Genotype, γ‐Promoters, and β‐LCR Hypersensitive Sites 2 and 4 in Italian Patients. *American Journal of Hematology*.

[19] Khan J., Ahmad N., Siraj S., Hoti N. (2015). Genetic Determinants of β-Thalassemia Intermedia in Pakistan. *Hemoglobin*.

[20] Wainscoat J. S., Kanavakis E., Wood W. G. (1983). Thalassaemia Intermedia in Cyprus: The Interaction of α and β Thalassaemia. *British Journal of Haematology*.

[21] Galanello R., Dessi E., Melis M. (1989). Molecular Analysis of Beta Zero-Thalassemia Intermedia in Sardinia. *Blood*.

[22] Viprakasit V., Ekwattanakit S. (2019). Genetic Modifiers in β-Thalassemia. *Hemoglobin*.

[23] Fucharoen S., Weatherall D. J. (2012). The Hemoglobin E Thalassemias. *CSH Perspectives in Medicine*.

[24] Pornprasert S., Thichak S., Kongthai K., Wangchauy C. (2018). Comparison of HbA2, E, F and Red Cell Parameters in Homozygous HbE With and Without α0-Thalassemia Trait. *Laboratory Medicine*.

[25] Charoenkwan P., Wanapirak C., Thanarattanakorn P. (2005). Hemoglobin E Levels in Double Heterozygotes of Hemoglobin E and SEA-Type Alpha-Thalassemia. *Southeast Asian Journal of Tropical Medicine and Public Health*.

[26] Sanchaisuriya K., Chirakul S., Srivorakun H. (2008). Effective Screening for Double Heterozygosity of Hb E/α0-Thalassemia. *Annals of Hematology*.

[27] Sharma A., Marwah S., Buxi G., Yadav R. (2013). Hemoglobin e Syndromes: Emerging Diagnostic Challenge in North India. *Indian Journal of Hematology and Blood Transfusion*.

[28] Munkongdee T., Chen P., Winichagoon P., Fucharoen S., Paiboonsukwong K. (2020). Update in Laboratory Diagnosis of Thalassemia. *Frontiers in Molecular Biosciences*.

[29] Yin X. ‐L., Wu Z. ‐K., Zhou X. Y. (2012). Co‐Inherited β‐Thalassemia Trait and HbH Disease: Clinical Characteristics and Interference in Diagnosis of Thalassemia by High‐Performance Liquid Chromatography. *International Journal of Laboratory Hematology*.

[30] Kaçmaz M., Aşıkovalı S. (2024). Genotype-Phenotype Correlation and Mutation Spectrum of HBB Gene in the Hatay Province of Turkey. *Cukurova Medical Journal*.

[31] Zimmermann M. B., Fucharoen S., Winichagoon P. (2008). Iron Metabolism in Heterozygotes for Hemoglobin E (HbE), α-Thalassemia 1, or β-Thalassemia and in Compound Heterozygotes for HbE/β-Thalassemia. *American Journal of Clinical Nutrition*.

[32] Chaudhry A. F., Malik Z., Shegos C. J. (2022). Rare Co-Inherited Alpha-Thalassemia Minor and Beta-Thalassemia Minor With Heterozygous H63D Mutation Mistaken as Iron Deficiency Anemia: A Case Report. *AME Case Reports*.

